# Comparison of Different Intubation Methods in Difficult Airways during Simulated Cardiopulmonary Resuscitation with Continuous Chest Compression: A Randomized Cross-Over Manikin Trial

**DOI:** 10.1155/2019/7306204

**Published:** 2019-08-20

**Authors:** Togay Evrin, Jacek Smereka, Damian Gorczyca, Szymon Bialka, Jerzy Robert Ladny, Burak Katipoglu, Lukasz Szarpak

**Affiliations:** ^1^Department of Emergency Medicine, Ufuk University Medical Faculty, Dr Ridvan Ege Education and Research Hospital, 06520 Cankaya, Ankara, Turkey; ^2^Department of Emergency Medical Service, Wroclaw Medical University, Parkowa 34, Wroclaw, Poland; ^3^Medical Simulation Center, Lazarski University, Swieradowska 43, 02-662 Warsaw, Poland; ^4^Department of Anaesthesiology and Intensive Care, Medical University of Silesia, 3-go Maja 13-15, 41-800 Zabrze, Poland; ^5^Department of Emergency Medicine and Disaster, Medical University Bialystok, Szpitalna 37, 15-295 Bialystok, Poland

## Abstract

**Introduction:**

Airway management is one of key elements of resuscitation. Endotracheal intubation is still considered the gold standard for airway management during resuscitation.

**Aim:**

The aim of the study was to compare success rates and intubation time of different endotracheal intubation methods during emergency intubation with difficult airways in the conditions of cardiopulmonary resuscitation in a standardized manikin model.

**Methods:**

The study was designed as a prospective, randomized, cross-over simulation study. It involved 46 paramedics with at least 5 years of experience in Emergency Medical Service. The participants performed endotracheal intubation under difficult airway conditions during continuous chest compression, implemented with the LUCAS3 chest compression system. Three methods of tracheal intubation were applied: (1) standard Macintosh laryngoscope without a bougie stylet; (2) standard laryngoscope and a standard bougie stylet; (3) standard laryngoscope and a new bougie stylet.

**Results:**

The overall intubation success rate was 100% in the standard bougie and new bougie groups and lower (86.9%) when no bougie stylet was used (P=0.028). The intubation success rate with the 1^st^ attempt equalled 91.3% for the new bougie group, 73.9% for standard bougie, and only 23.9% in the no-bougie group. The median intubation time was shortest in the new bougie group, where it amounted to 29 s (interquartile range [IQR]: 25–38); the time equalled 38s (IQR:31–44.5) in the standard bougie group and 47.5s (IQR:36–58) in the no-bougie group. The ease of use was lowest in the no-bougie group (85, IQR:63–88), average in the standard bougie group (44, IQR:30–51), and highest in the new bougie stylet group (32, IQR:19–41).

**Conclusion:**

In this manikin-based study, paramedics were able to perform endotracheal intubation with higher efficacy and in a shorter time using the new bougie stylet as compared with the standard bougie stylet.

## 1. Introduction

The European Resuscitation Council (ERC) guidelines recommend endotracheal intubation as the most reliable airway management [1–3]. The advantages of endotracheal intubation comprise isolation of the airway from the oesophagus, dramatic reduction of the risk of aspiration, reliability of ventilation, possibility of performing airway suctioning with secretion removal, and lack of significant leaks at excessive pressures generated during chest compression and disadvantages include in particular hyperventilation and excessive inflation [4–6].

There are a number of limitations and problems related to endotracheal intubation [1, 7]. The main issue is the need of adequate experience [8–10]. Patient intubation in difficult airways requires special skills, availability of appropriate equipment, and proper assessment of the clinical situation and priorities [2, 11]. Nevertheless, besides the lack of sufficient skills and experience, the main obstacles include such specific situations as a patient with a full stomach, a patient with cervical spine immobilization, a patient with trauma, and difficult airway due to anatomical conditions or pathological changes [12–14].

A special issue is endotracheal intubation during cardiopulmonary resuscitation. The ERC 2015 guidelines recommend that endotracheal intubation should be performed during chest compression, which may be interrupted for no more than 5 s [1, 15]. The stress associated with serious emergencies, such as cardiac arrest, makes some endotracheal intubations, especially those carried out by less experienced medical personnel, technically more difficult [16, 17] and time pressure is an additional challenge [18]. Much progress in intubation has been made since the introduction of intubation stylets, in particular the flexible bougie stylet [19, 20]. These stylets facilitate endotracheal intubation when laryngeal entry is poorly visible or completely invisible.

The aim of this study was to compare success rates and intubation time of different endotracheal intubation methods during emergency intubation with difficult airways in the conditions of cardiopulmonary resuscitation in a standardized manikin model.

## 2. Material and Methods

The study was approved by the Institutional Review Board of the Polish Society of Disaster Medicine (approval No.: 23.04.2019.IRB). Written informed consent was obtained from all participants. The study was designed as a prospective, randomized, cross-over simulation trial.

### 2.1. Study Population

The participants were recruited from among those working within the National Emergency Medical Service in Poland; 46 paramedics with at least 5-year work experience were included. All participants were routinely involved in the management and initial treatment of cardiac arrest patients in prehospital settings. This was single blinded study and the results were blinded at the stage of results analysis.

### 2.2. Simulation of the Scenario

A Resusci Anne Simulator (Laerdal, Stavanger, Norway) was used to simulate a patient with cardiac arrest requiring advanced life support, including endotracheal intubation. The simulator was placed on a flat surface in a brightly lit room. In order to standardize the difficulties resulting from continuous chest compression, a LUCAS3 system (Stryker, Kalamazoo, MI, USA) was applied. Difficult airways were achieved by filling the tongue with air to obtain Cormack-Lehane grade 3.

### 2.3. Devices

Before starting the study, all participants took part in a 30-minute training course on airway management. At the end of the theoretical training, the instructor, experienced in endotracheal intubation, demonstrated correct intubation with the use of the tested techniques. Then, the paramedics had the opportunity to participate in 10-minute practical workshop on endotracheal intubation, during which they practised intubation with all tested devices.

**Figure 1:**
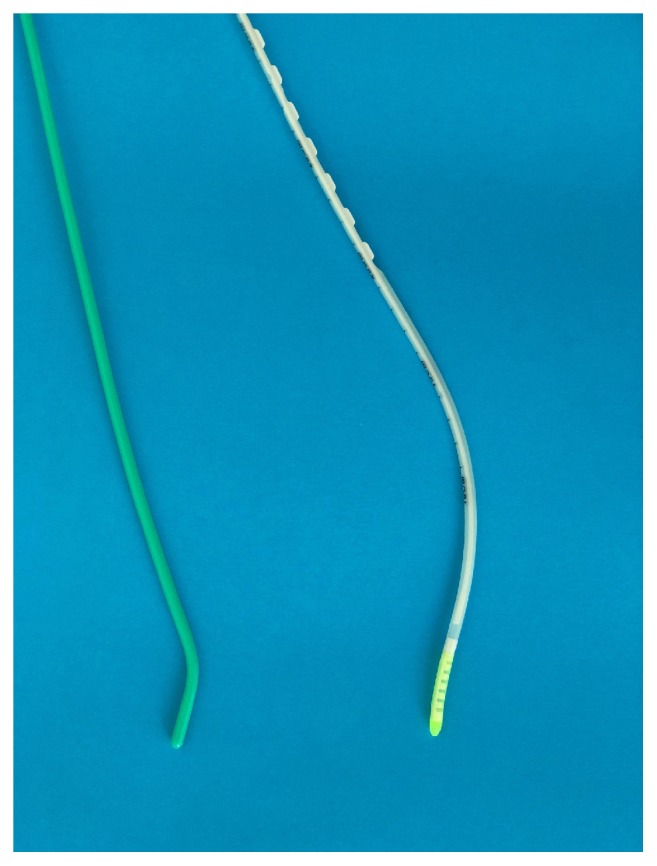
(a) Standard bougie stylet; (b) the new flexible tip bougie catheter.

Three intubation techniques were applied in the study:

- standard Macintosh laryngoscope with blade No. 3 without a bougie stylet;
- standard Macintosh laryngoscope with blade No. 3 and a standard bougie stylet (SUMI, Sulejowek, Poland; Figure 1(a));
- standard Macintosh laryngoscope with blade No. 3 and a new bougie stylet (MDSS GmbH, Hannover, Germany; Figure 1(b)).

**Figure 2:**
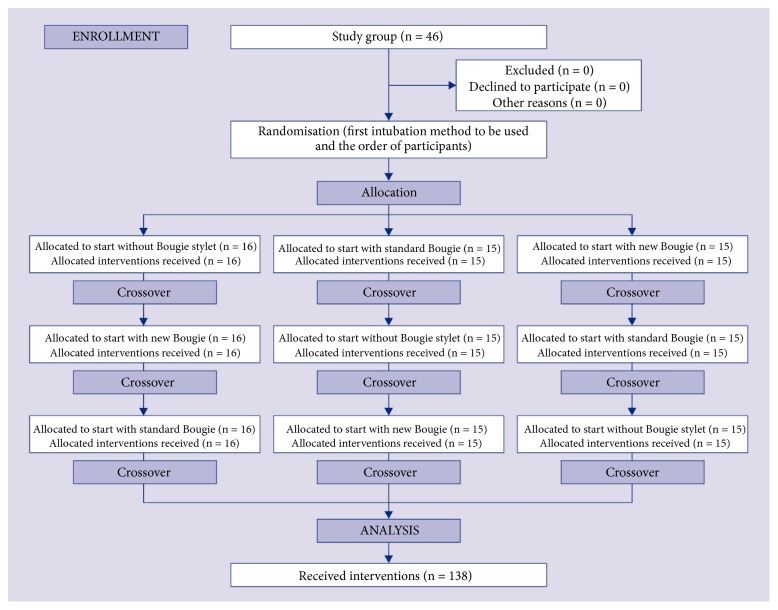
Randomization flow chart.

Endotracheal intubation was performed with continuous chest compression. Each participant had a maximum of three attempts to intubate with each technique. Randomization was done by using ResearchRandomizer software. A detailed randomization procedure is presented in the Figure 2.

### 2.4. Measurements

Successful endotracheal intubation was confirmed by a blinded to the study arms researcher when it was possible to ventilate the manikin lungs with a self-inflating bag connected to the endotracheal tube. The following criteria were defined for a failed intubation: more than 3 unsuccessful intubation attempts, intubation procedure time exceeding 120 s, or unrecognized oesophageal intubation.

The secondary outcome was time to intubation, defined as the time from inserting the laryngoscope blade between the teeth to the first manual ventilation of the manikin's lungs.

The participants provided their subjective opinions about the ease of each intubation method by pointing at a score on a visual analogue scale ranging 1–100, with 1 meaning “extremely easy” to 100 standing for “extremely difficult”.

### 2.5. Statistical Analysis

The sample size was calculated with the G*∗*Power 3.1 software with a 2-tailed t-test (Cohen's d: 0.8, alpha error: 0.05, power: 0.95). According to the calculation, a minimum of 45 participants were necessary.

All analyses were performed with the Statistica 13.3 EN statistical package (Tibco Inc., Tulsa, OK, USA). The value of P < 0.05 was considered significant. Data are presented as number (percentage), mean ± standard deviation, or median (interquartile range [IQR]), as appropriate. Nonparametric tests were used for the data that did not have a normal distribution. All statistical tests were 2-sided.

The Wilcoxon test for paired observations served to compare the different times and to determine the statistical difference for each group. McNemar test was applied to assess the differences in intubation success rates. The score for the ease of intubation was evaluated with the Stuart-Maxwell test.

## 3. Results

The overall intubation success rate was 100% in the standard bougie and new bougie groups and lower (86.9%) when no bougie stylet was used; the difference was statistically significant (P = 0.028 for standard and new bougie groups compared with the no-bougie stylet group; Table 1).

The intubation success rate with the 1^st^ attempt equalled 91.3% for the new bougie group, 73.9% for standard bougie, and only 23.9% in the no-bougie group; the differences were statistically significant for all comparisons. In the 2^nd^ attempt, the intubation success rate was 8.7% for the new bougie group, 26.1% for standard bougie, and only 43.4% in the no-bougie group. The 3^rd^ intubation attempt was necessary only for the no-bougie group, with the success rate of 19.6%.

**Figure 3:**
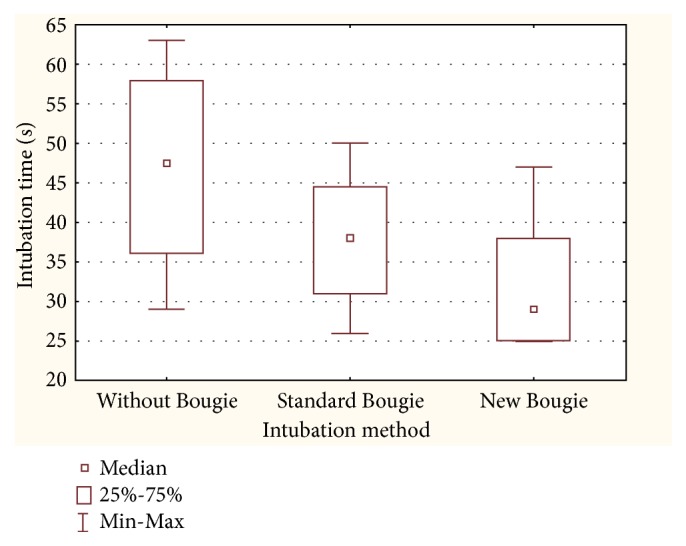
Median intubation time.

The median intubation time was shortest in the new bougie group, where it amounted to 29 s (interquartile range [IQR]: 25–38); the time equalled 38 s (IQR: 31–44.5) in the standard bougie group and 47.5 s (IQR: 36–58) in the no-bougie group (Figure 3). All these differences turned out statistically significant.

**Figure 4:**
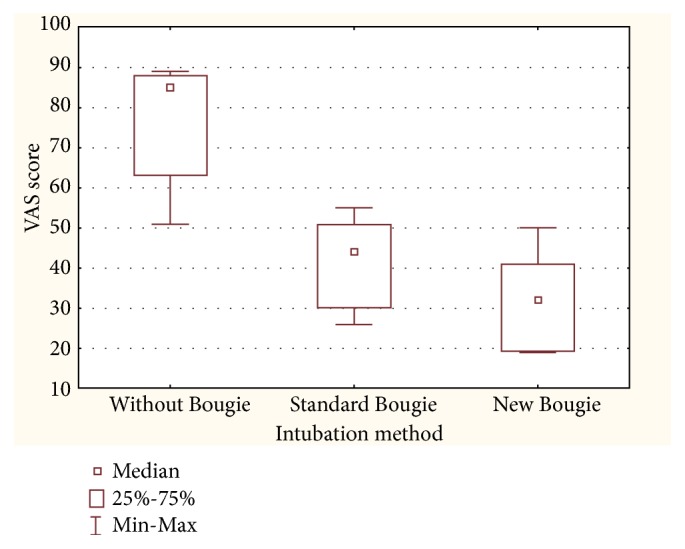
Ease of use scale.

The ease of use was lowest in the no-bougie group (85, IQR: 63–88), average in the standard bougie group (44, IQR: 30–51), and highest in the new bougie stylet group (32, IQR: 19–41). All these differences were statistically significant (Figure 4).

## 4. Discussion

The study showed that the use of the new bougie stylet was associated with a shorter intubation procedure in difficult airway scenario, as well as higher efficacy of the first intubation attempt. Airway management in emergency medicine is one of the basic elements of the treatment of patients with cardiac arrest [21–23]. Intubating the patient allows to perform asynchronous cardiopulmonary resuscitation and thus—in accordance with the ERC and American Heart Association (AHA) guidelines—to minimize interruptions in chest compressions [24, 25].

The present study shows that the new bougie stylet is superior to the standard stylet. This applies to both the effectiveness of the first intubation attempt and the time taken to complete the procedure. These results remain in line with those observed in a study by Brunckhorst et al. [26]. Research carried out by Driver et al. [19] has also shown that the use of a bougie compared with an endotracheal tube with a stylet resulted in a significantly higher first-attempt intubation success among patients undergoing emergency endotracheal intubation. Also, a study by Komasawa et al. suggests that a gum-elastic bougie facilitates tracheal intubation during chest compressions performed by novice physicians in adult simulations [27]. Lastly, Sheu et al. indicate that the use of a bougie stylet compared with a standard endotracheal stylet during endotracheal intubation of patients under emergency medical conditions was associated with higher efficacy of the first intubation attempt [28].

The effectiveness of the first attempt of intubation without a bougie stylet in the study was 23.9%, while the total effectiveness of intubation equalled 86.9%. In a manikin simulation study conducted by Szarpak et al. [29], the first attempt of intubation with a Macintosh laryngoscope in difficult airway conditions with continuous mechanical chest compression turned out effective in 17% and the total intubation efficiency was 75%. Low efficacy of the first intubation attempt in difficult airway conditions during resuscitation has been also reported in paediatric patients. Smereka et al. showed a 29.1% efficacy of the first intubation attempt with Miller's blade laryngoscope in difficult airway conditions [30]. Suzuki et al., on the other hand, reported the efficacy of direct laryngoscopy in emergency medicine patients at the level of 58% [31]. Such low intubation efficiency may be associated with two issues: difficult airways and challenges created by continuous chest compression during endotracheal intubation [32, 33]. Numerous studies have shown that the efficacy of direct laryngoscopy intubation in difficult airway conditions may be limited. To facilitate intubation, as demonstrated in this case, videolaryngoscopes may be an alternative [34–36], or the intubator can use a bougie stylet for difficult intubation. The second parameter that may reduce the effectiveness of intubation may be the fact that intubation is performed during resuscitation procedures, where chest compressions are implemented in parallel: moving the patient may make it difficult to visualize the vocal structures [27, 37].

The cardiopulmonary resuscitation guidelines by both ERC and AHA recommend to minimize interruptions in chest compressions and to perform endotracheal intubation during chest compressions. In this study, intubation with a Macintosh laryngoscope lasted 47.5 s without a stylet and 38 s with a standard bougie stylet.

The time was the shortest (29 s) in the case of a new bougie stylet. Brazil et al. demonstrated that success rates for novice practitioners using a gum-elastic bougie were high even after limited instruction and practice [38]: among junior doctors working in an emergency department, the intubation time with a bougie stylet was 24.18 s (21.45–27.95). This shorter intubation time reported by Brazil et al. compared to our findings could be related to the challenge of intubating with ongoing mechanical chest compressions.

Among the studied methods of endotracheal intubation, the one with a new bougie stylet turned out the simplest; intubation with a standard laryngoscope with a Macintosh blade was the most difficult. In the case of difficult airway, when it is impossible to optimally visualize the entrance to the glottis, difficult intubation stylets can be helpful, allowing the intubation tube to be inserted into the trachea without the need to visualize it. Intubation with a Miller or Macintosh blade laryngoscope was insufficiently effective under difficult airway conditions and the duration of the procedure was prolonged as compared with intubation with the use of a stylet or videolaryngoscope [39–41].

The study has both limitations and strengths. Among the limitations, one could mention that the study was conducted under medical simulation conditions and not during real rescue operations; however, this choice was deliberate and dictated by the fact that in emergency medicine, the conduct of a randomized cross-over study could adversely affect the patients' health. Another limitation is the inclusion of paramedics only, but it is their skills and medical equipment that is relied on in the prehospital environment; the search for new, more effective methods of airway management is crucial in this respect. Among the strengths of the study there are, among others, its randomized cross-over nature, as well as the fact that medical simulation allows to fully standardize medical procedure conditions. Another strong point was the evaluation of one of the latest bougie stylets and its confrontation with two other endotracheal intubation methods.

## 5. Conclusion

In the presented manikin-based study, paramedics were able to perform endotracheal intubation with higher efficacy and in a shorter time using the new bougie stylet as compared with the standard bougie stylet.

## Figures and Tables

**Table 1:** Intubation success rates, time, and ease in the analysed groups.

Parameter	Intubation method			p-value
	*Without Bougie (A)*	*Standard Bougie (B)*	*New Bougie (C)*	
Overall intubation success rate, n (%)	40 (86.9%)	46 (100%)	46 (100%)	A vs. B = 0.028
				A vs. C = 0.028
				B vs. C = NS

Intubation success rate, n (%)				A vs. B <0.001
1^st^ attempt	11 (23.9%)	34 (73.9%)	42 (91.3%)	A vs. C <0.001
2^nd^ attempt	20 (43.4%)	12 (26.1%)	4 (8.7%)	B vs. C = 0.047
3^rd^ attempt	9 (19.6%)	-	-	

Intubation time, s (IQR)	47.5 (36-58)	38 (31-44.5)	29 (25-38)	A vs. B = 0.015
				A vs. C = 0.002
				B vs. C =0.038


Ease of use, VAS score (IQR)	85 (63-88)	44 (30-51)	32 (19-41)	A vs. B <0.001
				A vs. C <0.001
				B vs. C = 0.043


NS: not significant; IQR: interquartile range; VAS: visual analogue scale, from 1 (extremely easy) to 100 (extremely difficult).

## Data Availability

The data used to support the findings of this study are available from the corresponding author upon request.
